# A case-control study to identify risk factors for adult-onset idiopathic megaoesophagus in Australian dogs, 2017–2018

**DOI:** 10.1186/s12917-020-02376-6

**Published:** 2020-05-24

**Authors:** M. Renwick, M. A. Stevenson, A. Wiethoelter, C. Mansfield

**Affiliations:** Faculty of Veterinary and Agricultural Sciences, The University of Melbourne, Parkville, Victoria 3010 Australia

**Keywords:** Canine megaoesophagus, Epidemiology, Case-control study, Outbreak investigation

## Abstract

**Background:**

Epidemiological investigations were carried out following detection of an outbreak of megaoesophagus in Victorian Police working dogs in early 2018 and an increase in the number of canine megaoesophagus cases reported by companion animal veterinarians in Eastern Australia starting in late 2017. VetCompass Australia data were used to quantify the incidence of canine megaoesophagus for the period January 2012 to February 2018 and a matched case-control study carried out to identify individual animal risk factors for canine megaoesophagus in 2017–2018.

**Results:**

There was a 7-fold increase in the incidence rate of canine megaoesophagus from 2014 (0.11 [95% CI 0.02 to 0.58] cases per 100,000 dogs per day) to 2018 (0.82 [95% CI 0.19 to 4.2] cases per 100,000 dogs per day). Since 2013, the incidence of megaoesophagus in Australia has shown a seasonal pattern, with greater numbers of cases diagnosed during the warmer months of the year. In the case-control study, use of Mars Petcare Advance Dermocare as a source of food was 325 (95% CI 64 to 1644) times greater for cases, compared with controls.

**Conclusions:**

Our analyses provide evidence that the feeding of Advance Dermocare was responsible for the majority of cases in the outbreak of megaoesophagus in Eastern Australia in 2017–2018. The increase in the incidence rate of megaoesophagus in Australia since 2014–2015 warrants further investigation.

## Background

Megaoesophagus is a disorder characterised by a hypomotile, dilated oesophagus; and may be congenital or acquired, idiopathic or secondary to other disease [1]. Acquired megaoesophagus in dogs is most commonly idiopathic [2]. Conditions associated with acquired megaoesophagus include myasthenia gravis, systemic myopathies, hypoadrenocorticism, lead toxicity, dysautonomia, severe oesophagitis, and systemic lupus erythematosus [3]. Hypothyroidism has been cited as an underlying cause of acquired secondary megaoesophagus [4], yet in a case-control study hypothyroidism was not associated with megaoesophagus [5].

Chronic regurgitation is a characteristic clinical sign of canine idiopathic megaoesophagus (IMO) and affected dogs frequently lose body condition and develop aspiration pneumonia [1]. Cervical and thoracic plain and contrast radiography, including fluoroscopy in non-anaesthetised patients is used to diagnose megaoesophagus [1]. Further clinical investigations are required to exclude causes of acquired megaoesophagus before a diagnosis of IMO can be made [1]. Treatment of IMO is palliative and involves frequent feeding of food in an upright position, and medications for associated aspiration pneumonia (e.g. amoxicillin-clavulonate) and oesophagitis (e.g. omeprazole) as required [1]. The prognosis for IMO in dogs is poor, with owners requesting euthanasia because of chronic cachexia and aspiration pneumonia [1].

During late 2017 regurgitation soon after the consumption of food was observed in six dogs owned and trained by the Victoria (Australia) Police Dog Squad. From January 2018, this group of dogs were investigated at the U-Vet Werribee Animal Hospital at the University of Melbourne. All six dogs were confirmed to have IMO by radiology, fluoroscopy and additional testing as indicated (e.g. adrenal function testing, endoscopy, electromyography and anti-acetylcholine receptor antibody concentration). After completion of these investigations, the commercial dry diet routinely fed to Victoria Police Dog Squad dogs (Advance Dermocare) was hypothesised to be the most likely causal factor connecting the identified IMO cases.

Following additional reports of IMO in pet dogs on social media and to the Australian Veterinary Association, the commercial dry food diet (Advance Dermocare) was recalled from the market by its manufacturer, Mars Petcare Australia. Subsequently, through notifications by veterinarians using the PetFAST system (a voluntary joint initiative of the Australian Veterinary Association and the Pet Food Industry Association of Australia to monitor health problems in dogs and cats suspected of being associated with pet food), communication with those involved in monitoring the Mar Petcare hotline and direct communication, further cases were logged with the University of Melbourne investigators.

To the best of our knowledge, quantitative estimates of the incidence of IMO in Australia or other countries have not been reported. We are aware of only one other outbreak of IMO that occurred in Latvia between 2014 and 2016. In this outbreak, a case-control study showed that the use of a specific (but different) dry commercial dog food was more commonly fed to IMO cases, compared with controls (Ilze Matīse, pers. comm., 25 April 2018).

To deal with what appeared to be an emerging disease problem, an IMO working group was formed in April 2018 comprised of clinicians and veterinary epidemiologists from the Faculty of Veterinary and Agricultural Sciences at the University of Melbourne. The mandate of this group was to coordinate activities related to learning more about the epidemiology of IMO in Australian dogs, which might then contribute to the development of evidence-based control strategies. This paper provides a description of the two main investigatory activities carried out by the IMO working group: (1) provision of estimates of the incidence of canine megaoesophagus over time to provide quantitative evidence that there was an increase in the frequency of IMO throughout Australia in 2017–2018, compared with previous years; and (2) a matched case-control study to identify individual animal risk factors for IMO.

## Results

For the period January 2012 to February 2018 (inclusive) details of individual veterinary consultations for 1,160,940 dogs were available from VetCompass Australia. Of this group, 1312 dogs were over 6 months of age and had the term ‘megaoesophagus’ (or one of its variants) recorded on at least one occasion in their clinical records.

**Fig. 1:**
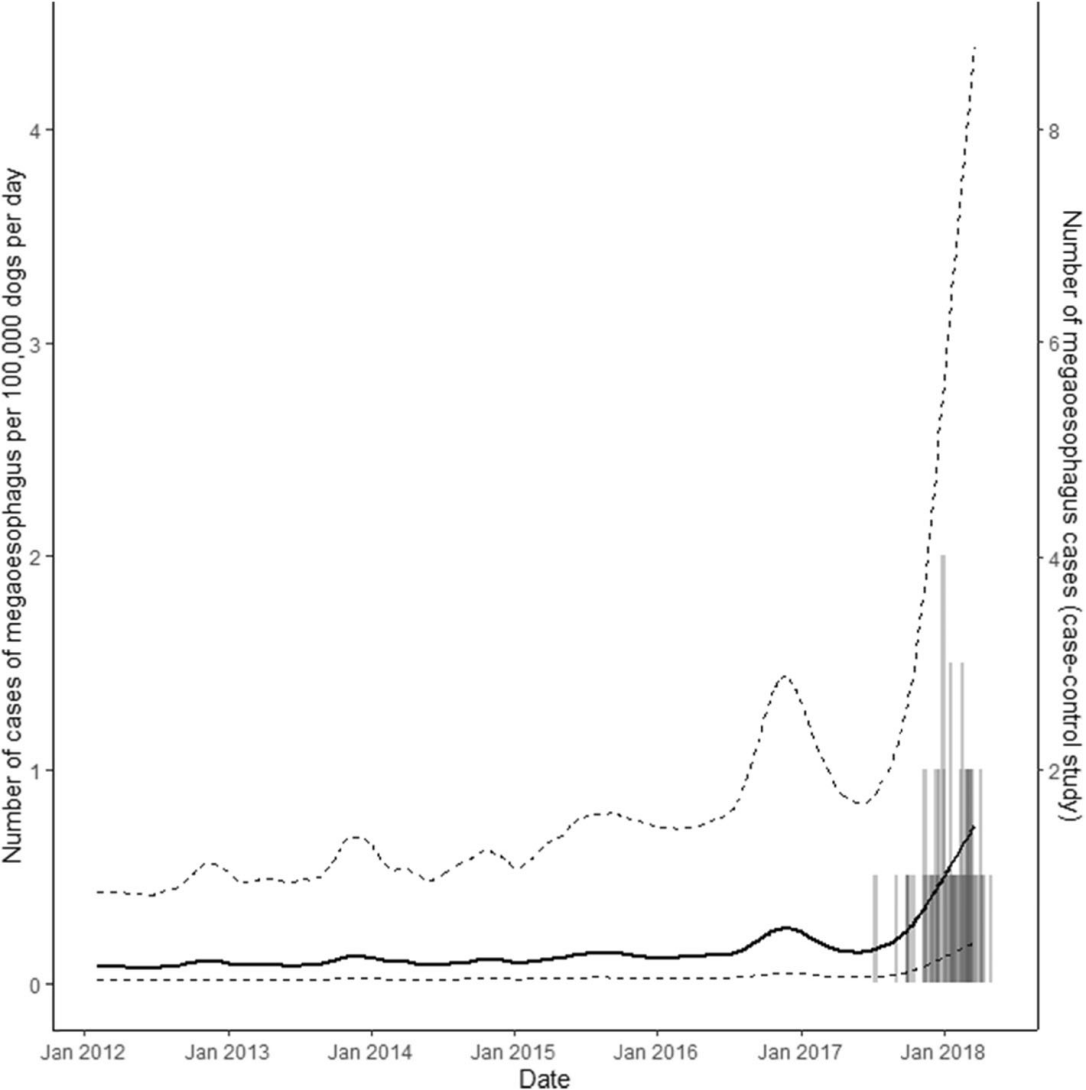
Line plot showing the incidence rate of canine megaoesophagus (expressed as the number of cases per 100,000 dogs per day) 1 January 2012 to 15 February 2018. The solid line shows the point estimate of megaoesophagus incidence rate as a function of calendar date. The lower and upper dashed lines show the lower and upper 95% confidence intervals around the incidence rate estimates. Superimposed on this plot is a frequency histogram showing date of onset of clinical signs for megaoesophagus cases used in the case-control study

Figure 1 is a line plot showing the number of cases of megaoesophagus per 100,000 dogs per day (and its 95% CI) as a function of calendar time for the period 1 January 2012 to 15 February 2018 (inclusive). Superimposed on this plot on the secondary vertical axis is a frequency histogram showing counts of the date of onset of clinical signs of case-control study IMO cases. There was a 7-fold increase in the incidence rate of canine megaoesophagus from 2014 to 2018. In 2014 the mean incidence rate of megaoesophagus was 0.11 (95% CI 0.02 to 0.58) cases per 100,000 dogs per day; in 2018 the mean incidence rate of megaoesophagus had increased to 0.82 (95% CI 0.19 to 4.2) cases per 100,000 dogs per day. Since 2013 the incidence of megaoesophagus in Australia has shown a seasonal pattern, with greater numbers of cases diagnosed during the warmer months of the year, as indicated by the upper bounds of the 95% confidence interval of the IMO incidence rate as a function of calendar time.

Questionnaires were sent to the owners of 184 possible IMO cases. Twenty-five questionnaires were subsequently removed from the analysis: seven were found not to be IMO; four cases were not idiopathic (three myasthenia gravis and one hypothyroid); two were congenital megaoesophagus; one presented with atypical signs putting its diagnosis in doubt; seven had an onset of clinical signs that was not within the required time frame for the study; one veterinarian requested that we send the questionnaire to the referral veterinarian; two veterinarians requested that the questionnaire be re-sent; and one was a duplicate. Seventy-seven of the 159 (48%) remaining questionnaires were for dogs that were subsequently identified as confirmed cases of IMO. A total of 121 questionnaires were completed for the 237 controls, a response rate of 51%.

Questionnaire details from 77 cases and 121 controls were available for analysis, a ratio of approximately 1:1.6. For IMO cases the median date of onset of clinical signs was 27 January 2018. The range of date of onset of clinical signs was 11 July 2017 to 1 May 2018. Forty-nine cases (64%) were male (six entire and 43 neutered) and 28 cases (36%) were female (two entire and 26 neutered). Cases had a median age of 6 years and 8 months (IQR [interquartile range] 4 years 7 months, 8 years 8 months) at the time of diagnosis and a median bodyweight of 30 (IQR 25, 39) kg at the time of diagnosis.

Table 1 lists the unconditional associations between case-control status and each of the putative explanatory variables with *p* < 0.20. Breeds over-represented in the cases included Dalmatians, Golden Retrievers and Labradors and, to a lesser extent, German Shepherds and German Shorthaired Pointers. A greater variety of diets were offered to control dogs compared with case dogs. A greater proportion of case dogs were fed Mars Petcare Advance Dermocare compared with control dogs. A smaller proportion of case dogs were fed canned (wet) commercial diets, semi-dry commercial diets, scraps or ‘other’ diets compared with controls. In this study we use the term ‘other diet’ to refer to home-made diets commonly comprised of a variety of meats and vegetables. Although data were collected on manufacturer, brand and the specific name of all commercial feeds offered, only results for commercial dry diets are presented in Table 1.

**Table 1:** Risk factors for idiopathic canine megaoesophagus in Australia, 2017–2018. Unconditional associations between case-control status and putative explanatory variables with *p* < 0.20

Variable	Number of		OR (95% CI)	***p***-value
	Cases	Controls		
Breed:				
Labrador	14	7	7.1 (2.4 to 21)	< 0.01
Golden Retriever	11	2	19 (3.8 to 99)	< 0.01
Dalmatian	9	2	16 (3.0 to 83)	< 0.01
German Shepherd	5	3	5.9 (1.2 to 28)	0.02
Other purebred	25	61	1.4 (0.67 to 3.1)	0.34
Crossbreed	13	46	Reference	
Gender:				
Male	49	61	1.7 (0.94 to 3.0)	0.08
Female	28	59	Reference	
Not reported	0	1		
Bodyweight (kg):				
< 10	2	43	Reference	
≥ 10 to 25	21	40	11 (2.5 to 51)	< 0.01
≥ 25 to 30	15	13	25 (5.0 to 120)	< 0.01
≥ 30	39	18	47 (10 to 210)	< 0.01
Not recorded	0	7		
Age (years):				
< 3	6	29	Reference	
≥ 3 to 7	34	41	4.0 (1.5 to 11)	< 0.01
≥ 7 to 10	23	15	7.4 (2.5 to 22)	< 0.01
≥ 10	8	28	1.4 (0.42 to 4.5)	0.59
Not recorded	6	8		
Dry food diet 100%:				
Yes	57	22	13 (6.4, 27)	< 0.01
No	17	86	Reference	
Not recorded	3	13		
Purina dry food:				
Yes	3	15	0.29 (0.08 to 1.0)	0.04
No	74	106	Reference	
Black Hawk dry food:				
Yes	0	8	0.09 (0.00 to 1.5) ^a^	0.04
No	77	113	Reference	
Mars dry food:				
Yes	70	47	16 (6.7 to 37)	< 0.01
No	7	74	Reference	
Hill’s dry food:				
Yes	3	14	0.31 (0.09 to 1.1)	0.06
No	74	107	Reference	
‘Other’ dry food: ^b^				
Yes	2	21	0.13 (0.03 to 0.56)	< 0.01
No	75	100	Reference	
Mars Advance Dermocare:				
Yes	68	2	450 (94 to 2100)	< 0.01
No	9	119	Reference	
Mars Advance other:				
Yes	1	16	0.09 (0.01 to 0.67)	< 0.01
No	76	105	Reference	
Mars Royal Canin:				
Yes	0	15	0.04 (0.0 to 0.75) ^a^	< 0.01
No	77	106	Reference	

^a^ Haldane-Anscombe corrected odds ratio

^b^ ‘Other dry food’ means brands of dry food excluding those specifically assessed in this study (Purina, Black Hawk, Hill’s and Mars Petcare)

The odds of exposure to a 100% dry food diet, a Mars dry food diet or a Mars Advance Dermocare diet was unconditionally greater in cases compared with controls (*p* < 0.01, Table 1). The odds of exposure to dry food brands excluding those specifically assessed in this study (Purina, Black Hawk, Hill’s and Mars Petcare) and other Mars Advance brands was unconditionally less in cases compared with controls (*p* < 0.01, Table 1).

In the final multivariable model, the odds of routinely being fed Mars Petcare Advance Dermocare in the 6 months prior to diagnosis was 325 (95% CI 64 to 1644) times greater for case dogs, compared with control dogs (Table 2). Five kilogram increases in bodyweight was associated with a 1.3 (95% CI 1.1 to 1.6) times increase in the odds of developing IMO (Table 2). The area under the ROC curve for the predictions from our multivariable model was 0.97 indicating that the multivariable model had excellent ability to discriminate between case and control dogs.

**Table 2:** Risk factors canine megaoesophagus in Australia, 2017–2018. Estimated regression coefficients and their standard errors from a mixed-effects logistic regression model of risk factors for idiopathic canine megaoesophagus

Variable	Number of		Coefficient (SE)	***p-***value	OR (95% CI)
	Cases	Controls			
Intercept	77	121	−3.8210 (0.6808)	< 0.01	
Bodyweight (× 5 kg)	77	121	0.2748 (0.1044)	< 0.01	1.3 (1.1 to 1.6)
Advance Dermocare:					
Yes	68	2	5.7849 (0.8267)	< 0.01	325 (64 to 1644) ^a^
No	9	119	Reference		1.00
Random effect:			Variance ^b^		
Clinic			0.4652 (1.1498)		

^a^ Interpretation: For cases (dogs with IMO) the odds of exposure to Advance Dermocare was 325 (95% CI 64 to 1644) times the odds of exposure to Advance Dermocare in controls

^b^ Variance of the clinic-level random effect term

Fifty-five of the 68 (81%) case dog owners that fed Advance Dermocare reported that this brand comprised 100% of their dog’s diet. For the remainder of this group, Advance Dermocare comprised 75% of the diet (*n* = 13). A higher proportion of case dogs that were fed 100% Advance Dermocare were reported as deceased (20 of 55; 36%), compared with those that were fed less than 100% Advance Dermocare (3 of 13, 23%); the unconditional odds of death for dogs fed 100% Advance Dermocare was 1.9 (95% CI 0.47 to 7.7; *p* = 0.36) times the odds of being deceased for dogs fed less than 100% Advance Dermocare. Eleven of the 13 case dog owners who fed less than 100% Advance Dermocare provided details on the way it was fed with four mixing it with other foods, five feeding it separately to other foods and two using a combination of mixing and separate feeding.

## Discussion

Based on the marked increase in the incidence of megaoesophagus during late 2017 and early 2018 (Fig. 1), our inference is that by June 2017 the frequency of megaoesophagus in dogs had increased beyond that expected by chance and that an outbreak situation did, in fact, exist. Two features are noteworthy in Fig. 1. Firstly, while the incidence rate of megaoesophagus was relatively static at a mean of 0.08 cases per 100,000 dogs per day for the period January 2012 to 2016, the frequency of disease increased in late 2016 but this increase appears not to have been sufficient to generate a level of concern amongst veterinary practitioners to trigger an outbreak investigation response. Secondly, for the period 2012 to 2018 a seasonal pattern in the incidence rate of megaoesophagus is evident, with greater numbers of cases occurring during the warmer months of each year (October to December). We can only speculate regarding the reasons for this finding. Our first possible explanation is that if megaoesophagus was caused by a toxin present in commercial feed there may be seasonality in the contamination of commodity feed ingredient(s). Our second explanation (if there is no seasonal variation in ingredient contamination) is that the seasonal pattern of megaoesophagus incidence may be due to increases in the sale, and therefore consumption, of hypoallergenic canine diets during the warmer months of the year. A reduction in the incidence of megaoesophagus to less than (say) 0.08 cases per 100,000 dogs per day following recall of Advance Dermocare from the Australian market will provide some assurance that contamination was an issue only affecting Advance Dermocare whereas an incidence rate of greater than 0.08 cases per 100,000 dogs per day will provide indirect evidence that contamination (or otherwise) is an issue affecting other commercial dog foods, albeit at a lower level than that experienced by Advanced Dermocare. Samples of Advance Dermocare were tested by one of the authors (CM) for minerals and micronutrients (at Massey University, New Zealand), common food toxins, mycotoxins, neurotoxins and heavy metals (at the National Measurement Institute within the Department of Industry, Science, Energy and Resources, Australia and Agrifoods Australai). Despite testing for an extensive range of possible toxins, a single agent responsible for the Australian 2017–2018 IMO outbreak has not been identified.^1^ Comparison of the formulation used for the manufacture of Advanced Dermocare with that used by for the dog food hypothesised to be the cause of the Latvian megaoesophagus outbreak [6] and identification of similarities in either the ingredients used or the way they are processed would provide much needed insight for identifying the agent responsible for this preventable cause of debilitating canine illness.

The analyses presented in this paper demonstrate the enormous value of the VetCompass Australia initiative as a source of small animal surveillance data. Moving forward, a challenge for the Australian veterinary profession and pet food industries is to determine how best to use VetCompass data to develop proactive approaches to emerging food-borne disease detection, as opposed to the entirely reactive approach described in this study.

We identified a strong association between the feeding of Mars Petcare Advance Dermocare and the development of IMO. Dogs that were fed this diet were 325 (95% CI 64 to 1644) times more likely to develop idiopathic megaoesophagus compared with dogs fed other diets. Our unconditional analyses show that dogs fed a diet comprised of 100% Advance Dermocare was associated with a greater risk of death from IMO compared with those dogs where the diet was comprised of less than 100% Advance Dermocare, providing further support to the hypothesis that Advance Dermocare was the cause of this outbreak. Future investigations will document the survival of IMO cases arising from the 2017–2018 Australian outbreak and seek to determine why only some dogs in multi-dog households were affected, while others were spared.

Dalmatians, Golden Retrievers and Labradors were over-represented among cases compared with controls in this study. These are breeds susceptible to allergic skin disease [7] and would therefore be expected to be more likely to have been exposed to Advance Dermocare or other diets used in the management of allergic skin disease. Previous research in the United Kingdom determined the breed distribution of canine megaoesophagus. Of 71 cases, of which 54 were assessed as idiopathic, 20% were German Shepherds, 8% were Golden Retrievers, 8% were Great Danes, 6% were Labradors and 3% were Irish Setters (McBrearty, Ramsey et al. 2011). A similar distribution of affected breeds (Dobermans and Bernese Mountain Dogs, but also Labradors, German Shepherds and Golden Retrievers) was found in the 2016 Latvian outbreak [6]. Differences in the breeds represented in these studies may be due to breed preferences in each respective country. The similarities however suggest that the breed distribution seen in our study may not be entirely due to the increased chance of being fed Advance Dermocare but instead may be due to a breed predisposition to idiopathic canine megaoesophagus. It is also possible that dry food is used preferentially in larger breed dogs due to financial considerations.

Owners of 64 of the 70 case dogs that were fed Advance Dermocare reported that they routinely purchased feed in 15 kg bags; one owner purchased 8 kg bags; three owners did not provide an answer to this question and one owner reported buying 3 kg bags initially but then purchased a large bag after which their dog became ill approximately 2 weeks later. If only the large size bags were involved in this outbreak, this supports our finding that case dogs were heavier than control dogs in the bivariate analyses (Table 1). Our logistic regression model shows that odds of IMO increased by a factor of 1.3 (95% CI 1.1 to 1.6) for 5 kg increases in bodyweight, after accounting for the effect of feeding Advance Dermocare. We conclude from this analysis that dogs that were of heavier bodyweight were more likely to develop IMO although the relatively small number of cases for this study meant that there was insufficient statistical power to determine if bodyweight was a direct cause of IMO or if it was confounded by breed.

The media coverage that connected Advance Dermocare to IMO cases provided necessary communication with the dog-owning public and may well have prevented additional cases. Media coverage, however, could have introduced selection bias into the case-control study. Public awareness of the suspected cause of the outbreak could have made it more likely that both owners and vets of dogs that experienced megaoesophagus and were fed Advance Dermocare diet offered to participate in the study than those of dogs with megaoesophagus that had not been fed this diet. The investigators called for any cases of megaoesophagus during the study period to also be logged, through the Australian Veterinary Association and a large private veterinary group, Greencross Vets, in an attempt to address this potential bias.

As the diagnosis of IMO in dogs is a process of exclusion and not all cases had exhaustive diagnostic workups, there is the potential for misclassification bias in outcome status. Since the clinical signs of IMO, involving regurgitation soon after the consumption of food are characteristic of IMO it is our belief that the impact of this bias on the inferences made in this study is likely to be small.

## Conclusions

There was a 7-fold increase in the incidence rate of megaoesophagus in Australia in 2018 compared with 2014. A case-control study to identify risk factors for canine IMO in Australia between July 2017 and April 2018 found that cases were more likely to have been fed Advance Dermocare, compared with controls. We conclude that Advance Dermocare was the cause of this outbreak. Comparison of the formulation used for the manufacture of Advanced Dermocare with that used by for the dog food hypothesised to be the cause of the Latvian megaoesophagus outbreak would provide much needed insight for identifying the agent responsible for this preventable cause of debilitating canine illness.

## Methods

Two investigations are described in this paper. The first used data collected by Australian veterinary practices contributing de-identified individual animal clinical event data to the VetCompass Australia sentinel practice surveillance system [8]. The second investigation was a case-control study designed to identify individual animal management and feeding practices that rendered dogs during the period 1 July 2017 to 1 May 2018 at greater risk of being an incident case of IMO.

### Incidence of idiopathic megaoesophagus in Australia, 2012 to 2018

VetCompass Australia collates de-identified electronic patient record data from primary-care veterinary practices throughout Australia for epidemiological research [8]. Veterinary practices that participate in the VetCompass Australia project allow extraction of de-identified clinical records using appropriately configured practice management systems. Clinical records extracted by VetCompass include animal demographic details (species, breed, date of birth, sex, neuter status) as well as free-text clinical notes recorded by the attending veterinarian at the time of each consultation. We retrieved two data extracts from the VetCompass Australia database. The first comprised clinical records for dogs where the words ‘vomiting’, ‘regurgitation’, and ‘megaoesophagus’ (or their variants) were included in the animal’s clinical notes recorded by attending veterinarians at the time of consultation. The second data extract comprised the date of birth, the postcode of the owner’s home address and the month of the last consultation for all dogs listed in the VetCompass Australia database for the period January 2012 to February 2018 (inclusive).

We listed the clinical notes for each of the dogs included in the first data extract in turn and retrieved the animal’s date of birth and the earliest calendar date on which a consultation occurred where the term ‘megaoesophagus’ (or one of its variants) was recorded. Dogs less than 6 months of age were not included as these cases of megaoesophagus were considered more likely to be congenital. For the second data extract we retrieved the animal’s date of birth (set to the 15th day of each recorded month) and the calendar date of the last veterinary consultation. The two data sets were merged and a ‘status’ variable assigned to each dog, taking the value of 1 for those where the term megaoesophagus were mentioned in the clinical records (the first data extract) and 0 otherwise.

The incidence rate of IMO (that is, the number of IMO positive dogs per 100,000 dogs at risk per day) as a function of calendar date was estimated using survival analysis [9]. Here, the outcome of interest was the date on which IMO was first mentioned in an individual dog’s clinical notes. Dogs with a status of 0 (described above) were right censored on the date of their last recorded veterinary consultation. The incidence rate of IMO, interpreted as the probability of a dog being diagnosed with IMO per day given that it had remained free of disease to at least the specified point in time, was computed using the survival package [10] in R version 3.5.0 [11]. A time series plot was generated showing the incidence rate of IMO (the number of IMO positive dogs per 100,000 dogs at risk per day) and its 95% confidence interval as a function of calendar date.

### Case-control study

Possible, probable and confirmed cases of IMO for the case-control study were identified from three sources, as summarised in Table 3: (1) Mars Petcare; (2) the PetFAST reporting system; and (3) the University of Melbourne. The case definitions provided in Table 4 were used to distinguish between these groups. Attending veterinarians for each reported case were contacted and requested to complete a web-based questionnaire developed for the purpose of this study and administered using REDCap [12]. Clinical details, including an estimate of bodyweight before the onset of clinical signs, were collected for each reported case as well as details of exposure to hypothesised risk factors for megaoesophagus such as geographic location, vaccination history, use of internal and external antiparasitic treatments, housing and nutritional management (including the amount and type of feeds typically offered in the 6 months before the onset of clinical signs). Details of other dogs present in the household at the time of diagnosis were also collected. Possible, probable and confirmed cases of IMO were defined as ‘cases’ for the case-control study.

**Table 3:** Risk factors canine megaoesophagus in Australia, 2017–2018. Details of the different sources of data used to identify probable cases of megaoesophagus

Source	Details
Mars Petcare	Database of owners or vets of suspected MO cases shared with University of Melbourne.
Australian Veterinary Association	PetFAST reports.
University of Melbourne	Independent reporting to the U-Vet Werribee Animal Hospital, University of Melbourne from veterinarians and owners, including members of a megaoesophagus Facebook community. Reporting to the University of Melbourne responding to a call for cases sent by Australian Veterinary Association members and by internal communication within the Greencross Vets group of practices.

**Table 4:** Risk factors idiopathic canine megaoesophagus in Australia, 2017–2018. Definition of possible, probable and confirmed cases of idiopathic canine megaoesophagus used in this study

Case classification	Criteria
Possible	A dog with a history of chronic regurgitation ± clinical evidence of aspiration pneumonia.
Probable	Evidence of oesophageal dilatation on thoracic radiographs ± evidence of aspiration pneumonia; and Negative anti-acetylcholine receptor antibody assay.
Confirmed	Evidence of oesophageal dilation on thoracic radiographs (without sedation) or fluoroscopic swallowing study ± evidence of aspiration pneumonia; and Negative anti-acetylcholine receptor antibody assay; and Normal baseline cortisol/ACTH stimulation test; and Normal thyroid function.

Sample size calculations were carried out to determine the number of control dogs to recruit to determine if there was a statistically significant association between routine use of a given named brand of dog food and being an IMO case. A sample size of 70 case dogs and 70 control dogs was estimated using the Power and Sample Size Program version 3.0 [13]. These numbers were based on a case-control ratio of 1:1 with alpha of 0.05 and 80% power to detect an odds ratio (OR) of at least 3.0 for each of the exposures under investigation, assuming the prevalence of exposure amongst the controls was 0.3 and the correlation coefficient for exposure between matched cases and controls was 0.2.

Risk factors identified during the survey of cases directed the development of the questionnaire for the controls, which was comprised of nine questions regarding demographic and the use of routine prophylactic treatments plus a series of questions on nutritional management. A copy of the questionnaire can be viewed in Supplementary File 1. The control questionnaire was administered using REDCap and used an identical question format to those used in the case questionnaire. The time frame of interest for questions that related to nutritional management was the 6-month period before the date of presentation to the veterinarian. It was reasoned that asking questions about how dogs were typically fed over a relatively short time frame should minimise the impact of recall bias due to the unavoidable delay between the timing of the exposures that were being asked about and administration of the questionnaire.

For controls, veterinarians that had diagnosed a confirmed case of IMO were asked to select the three dogs that had been examined by any veterinarian in the same practice, immediately prior to the IMO case dog at first presentation for the condition [14]. If the case dog was seen too early in the day for this to be possible, the three dogs examined immediately after the IMO case were selected as candidate controls. The veterinarian that had diagnosed the IMO case was then asked to contact the owners of each candidate control dog to ask if they were interested in taking part in the study. If the owner of a candidate control dog agreed, the veterinarian either completed the web-based questionnaire together with the owner or sought consent from the owner to pass on their contact details to a member of the research team. If the owner did not agree to take part, the next dog examined at the clinic was selected as a replacement candidate control dog. The questionnaire was administered by telephone in most instances, but on some occasions, if the owner preferred, it was conducted by email. For all controls the responses to each question were transcribed into the questionnaire database using REDCap.

Data were downloaded from the REDCap database and statistical analyses carried out using R version 3.5.0 (R Core Team 2018) using the contributed epiR [15], survival [10] and R2MLwiN [16] packages. Bivariate (i.e. univariable) analyses were undertaken to select explanatory variables for multivariable modelling. The association between categorical exposure variables and dog case-control status was assessed using the odds ratio, with the Haldane-Anscombe correction [17, 18] used for comparisons where the frequency of any cell of the 2 × 2 table was equal to zero. The association between each of the continuously distributed exposure variables and dog case-control status was assessed using univariable logistic regression and the association between each of the categorical explanatory variables and IMO case status was assessed using the χ^2^ test. Where variables were correlated, the most biologically plausible of the two were selected for further analysis.

Exposure variables associated with a dog having IMO at an alpha level of less than 0.2 at the bivariate level were entered into a binary logistic regression model. For this analysis, the clinic from which case dogs and control dogs were selected (the matching variable) was included in the logistic regression model as a random effect term.

A backward elimination process was used to select explanatory variables associated with a dog being an IMO case. The significance of each explanatory variable in the model was assessed using the Wald test. Explanatory variables that were not statistically significant were removed from the model one at a time, beginning with the least significant, until the estimated regression coefficients for all the variables retained were significant at an alpha level of less than 0.05. The results of the final model are reported in terms of adjusted odds ratios for each explanatory variable. Assuming a causal relationship between a given exposure and IMO, an adjusted odds ratio (and its 95% confidence interval) of greater than 1 indicates that, after adjusting for other variables in the model, exposure to the explanatory variable increased the odds of a dog being an IMO case. An adjusted odds ratio (and its 95% CI) of less than 1 indicates that exposure to the explanatory variable was protective, and an odds ratio of 1 indicates that the variable was not associated with IMO risk.

A receiver operating characteristic (ROC) curve was constructed based on the IMO status of dogs predicted by the model. The area under the ROC curve, which ranges from zero to one, provided a measure of the model’s ability to discriminate IMO-positive and IMO-negative dogs. The greater the area under the ROC curve the better the model’s discriminatory power.

Use of the VetCompass Australia data for the purpose described in this study was approved by the University of Sydney’s Ethics Committee (Project: VetCompass Australia 2013/919). The case-control questionnaire survey was approved by the Human Ethics Committee of the University of Melbourne (Ethics ID 1851740.1). Informed verbal consent was obtained from all participants completing the questionnaire. Verbal, rather than written, consent was used because questionnaires were administered either by telephone or by electronic mail. Verbal consent was approved by the Human Ethics Committee of the University of Melbourne.

## Supplementary information

**Additional file 1.** Vet/owner questionnaire on canine megaoesophagus

## Data Availability

The data that support the findings of this study are available from VetCompass Australia but restrictions apply to the availability of these data, which were used under license for the current study and therefore not publicly available. Data are however available from the authors upon request and with permission of VetCompass Australia.

## Abbreviations

CI: Confidence interval
IMO: Idiopathic megaoesophagus
IQR: Interquartile range
ROC: Receiver operating characteristic (curve)

## Footnotes

1 URL: https://www.u-vet.com.au/news/megaesophagus-and-pet-food/megaoesophagus-and-pet-food-report14122018
