# Supplementary material for: A case-control study to identify risk factors for adult-onset idiopathic megaoesophagus in Australian dogs, 2017–2018
Source: BMC Vet Res. 2020 May 24;16:157. doi: 10.1186/s12917-020-02376-6 (PMC7247263; doi:10.1186/s12917-020-02376-6)
Supplement: Supplementary file 1 — Additional file 1. Vet/owner questionnaire on canine megaoesophagus [file 12917_2020_2376_MOESM1_ESM.docx]

**Vet/owner questionnaire on canine megaoesophagus**

Please complete the survey below.

Thank you!

# Section 1: Veterinarian details

Veterinarian first name: Veterinarian last name:

Veterinarian phone number (land line): Veterinarian phone number (mobile): Veterinarian email address:

Have your clients given permission for the investigators to contact them to obtain more details about their dog and its medical condition?


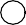
 Yes
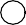
 No

# Section 2: Clinic - hospital details

Clinic - hospital name: Clinic - hospital address (1): Clinic - hospital address (2): Clinic - hospital suburb:

Clinic - hospital state: ACT


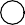

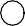

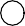

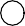

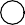

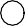

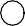


NSW NT QLD TAS VIC WA

Clinic - hospital postcode:

Clinic - hospital phone number (landline):

Clinic - hospital email address:

# Section 3: Owner details

Owner first name: Owner last name: Owner address (1):

Owner address (2): Owner suburb:

Owner state: ACT


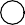

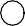

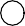

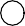

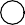

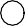

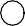


NSW NT QLD TAS VIC WA

Owner postcode:

Owner phone number (landline): Owner phone number (mobile): Owner email address:

Does the owner give the investigators permission to share their personal details with Mars Incorporated?

Does the owner give the investigators permission to share the clinical details of their dog with Mars Incorporated?

Does the owner give the investigators permission to share the dietary details (purchases etc) of their dog with Mars Incorporated?

Does the owner give the investigators permission to contact their veterinarian to obtain more details about their dog and its medical condition?

If 'no' has been selected for any of the above, does the owner give permission for information to be shared with the Australian Veterinary Association as part of the pet food recall working group?


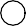
 Yes
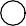
 No


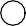
 Yes
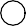
 No


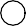
 Yes
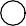
 No


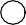
 Yes
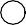
 No


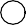
 Yes
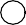
 No

# Section 4: Patient details

Patient name: Patient breed:

Patient gender:
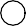
 Male
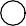
 Female

Patient reproductive status:
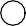
 Entire
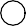
 Neutered

In what year did the dog enter the owner's household?

In (approximately) what month did the dog enter the Jan


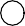

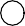

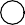

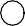

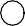

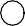

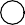

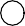

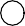

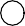

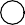

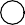


owner's household ? Feb

Mar Apr May Jun Jul Aug Sep Oct Nov Dec

How many months of age was the dog when it entered the owner's household?

Any previous (chronic) medical conditions?
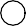
 Yes
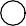
 No Please give details of previous chronic medical

conditions:

Flea treatment in the 12 months prior to presentation?


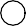
 Yes
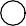
 No

Flea treatment details: Vaccinations in the 12 months prior to presentation?
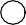
 Yes
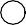
 No

Vaccination details:

Heartworm prophylaxis in the 12 months prior to presentation?


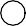
 Yes
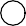
 No

Heartworm prophylaxis details:

Gastrointestinal parasite prophylaxis in the 12 months prior to presentation?


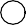
 Yes
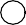
 No

Gastrointestinal parasite prophylaxis details:

# Section 5: Case details

Where was the dog routinely housed at the time of Outdoors in back yard diagnosis? Outdoors in kennel or run


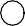

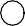

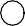


Indoors

Was this dog a working dog?
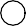
 Yes
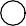
 No

If so, what type of work did the dog do? Police Army


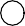

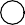

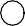

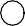

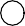

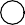


Guide/Hearing/Assistance Farm

Hunting Other

Were there any other dogs in the owner's household at the time of diagnosis?


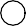
 Yes
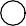
 No

Date of onset of clinical signs:

List the main clinical signs:

If the patient experienced regurgitation, did they Yes


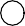

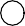


have any other gastrointestinal signs in the month No prior to onset of regurgitation?

If so, please give details of these gastrointestinal symptoms:

Was haematology performed as part of the case workup?
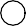
 Yes
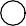
 No

Date of haematology:

Haematology details:

Was biochemistry performed as part of the case workup?


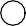
 Yes
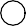
 No

Date of biochemistry:

Biochemistry details:

Was baseline cortisol measured as part of the case workup?


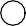
 Yes
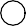
 No

Date of baseline cortisol:

Baseline cortisol details:

Were thyroid function tests carried out as part of the case workup?


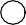
 Yes
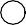
 No

Date of thyroid function:

Thyroid function details:

Was anti-acetylcholine antibody receptor concentration assessed as part of the case workup?


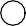
 Yes
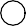
 No

Date of anti-acetylcholine antibody receptor concentration:

Anti-acetylcholine antibody receptor concentration details:

Were thoracic radiographs taken as part of the case workup?


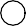
 Yes
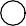
 No

Date of thoracic radiographs:

Was either sedation or general anaesthesia used to obtain these thoracic radiographs?


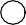
 Yes
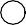
 No

Thoracic radiograph details:

Were contrast radiographs taken as part of the case workup?


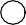
 Yes
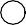
 No

Date of contrast radiographs:

Contrast radiograph details: Please upload file of radiograph (1) here, and

further radiographs below if necessary. Please upload file of radiograph (2) here.

Please upload file of radiograph (3) here. Please upload file of radiograph (4) here. Please upload file of radiograph (5) here. Please upload file of radiograph (6) here.

Briefly describe how this case was managed:

What is the status of the patient at the time of completion of this questionnaire?


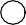
 Dead
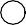
 Alive

What was the date of death?

Were any other dogs in the household affected?
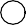
 Yes
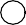
 No

(If the answer to this question is YES, please complete a separate questionnaire for each affected dog.)

# What type of food was typically fed in the 6 months prior to presentation? Use the check boxes to indicate the relative amount offered on a daily basis. The total amount offered must sum to 100%.

0% 25% 50% 75% 100%

Dry, commercial food:

Canned, commercial food (or pouch/tray):

Semi-dry commercial food:

Household scraps (including bones):

Other:

What was the name(s) of the manufacturer of the dry, Purina commercial food typically fed in the six months Woolworths

prior to presentation? Coles

Applaws Black Hawk Mars Petcare Hills

Other

What was the name(s) of the Purina dry food used? Pro Plan Purina One Supercoat Beneful Lucky Dog Bonnie Beyond Other

Please provide more details of the name(s) of the Purina dry food used, if necessary:

What was the name(s) of the Woolworths dry food used? Essentials

Other

Please provide more details of the name(s) of the Woolworths dry food used, if necessary:

What was the name(s) of the Coles dry food used? Complete Balance Other

Please provide more details of the name(s) of the Coles dry food used, if necessary:

What was the name(s) of the Applaws dry food used? It's All Good

Other

Please provide more details of the name(s) of the Applaws dry food used, if necessary:

What was the name(s) of the Black Hawk dry food used? Grain Free

Original Working Dog Other

Please provide more details of the name(s) of the Black Hawk dry food used, if necessary:

What was the name(s) of the Mars Petcare dry food Advance

used? Chum

Eukanuba Iams

My Dog Nutro Optimum Pedigree Royal Canin Other

Please provide more details of the name(s) of the Mars Petcare dry food used, if necessary:

What was the brand(s) of the Hills dry food used? Prescription Diet Science Diet

Science Diet VetEssentials Other

Please provide more details of the name(s) of the Hills dry food used, if necessary:

What was the brand(s) and name(s) of the other dry, commercial food used?

On approximately what date did the owner start using the type of dry, commercial food you named first?

Was a second dry, commercial pet food fed?
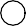
 Yes
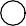
 No

On approximately what date did the owner start using the type of dry, commercial food you named second?

What was the name(s) of the manufacturer of the Purina

canned commercial food typically fed in the six Woolworths

months prior to presentation? Coles

Applaws Black Hawk Mars Petcare Hills

Other

What was the name(s) of the Purina canned food used? Proplan

Supercoat Beneful Other

Please provide more details of the name(s) of the Purina canned food used, if necessary:

What was the name(s) of the Woolworths canned food Essentials used? Other

Please provide more details of the name(s) of the Woolworths canned food used, if necessary:

What was the name(s) of the Coles canned food used? Smart Buy

Other

Please provide more details of the name(s) of the Coles canned food used, if necessary:

What was the name(s) of the Applaws canned food used? Natural Dog Food (cans/pouches)

Other

Please provide more details of the name(s) of the Applaws canned food used, if necessary:

What was the name(s) of the Black Hawk canned food Grain Free used? Other

Please provide more details of the name(s) of the Black Hawk canned food used, if necessary:

What was the name(s) of the Mars Petcare canned food Advance used? Chum

Eukanuba

My Dog (cans/pouches/trays) Nutro

Optimum (cans/trays) Pedigree

Royal Canin Other

Please provide more details of the name(s) of the Mars Petcare canned food used, if necessary:

What was the brand(s) of the Hills canned food used? Prescription Diet

Science Diet

Science Diet VetEssentials Other

Please provide more details of the name(s) of the Hills canned food used, if necessary:

What was the brand(s) and name(s) of the other canned, commercial food used?

On approximately what date did the owner start using the type of canned, commercial food you named first?

Was a second canned, commercial pet food fed?
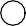
 Yes
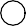
 No

On approximately what date did the owner start using the type of canned, commercial food you named

second?

What was the name(s) of the manufacturer of the semi-dry, commercial food typically fed in the six

months prior to presentation?

What was the name(s) of the semi-dry food used?

On approximately what date did the owner start using this type of semi-dry commercial food?

Please provide details of "other" diet used:

If dry commercial food was used, what size bags were routinely purchased?

3 kg 8 kg 15 kg other

Please state the usual bag size:

Where did the owner routinely buy their commercial Supermarket food? Pet store


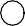

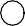

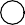

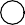

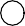


Veterinarian Multiple sources Other

Please provide details of other relevant dietary history in the 6 months prior to the diagnosis of

megaoesophagus:

What was the source of drinking water in the 6 months prior to the diagnosis of megaoesophagus?

Does the owner have samples of food remaining from prior to onset of clinical signs?

Does the owner have access to the batch numbers of commercial food purchased?


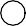
 Yes
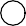
 No


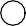
 Yes
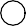
 No

Please provide batch numbers:
